# The genome sequence of a chalcid wasp, *Gastracanthus pulcherrimus *(Westwood, 1833)

**DOI:** 10.12688/wellcomeopenres.19764.1

**Published:** 2023-10-12

**Authors:** Gavin R. Broad, Chris Fletcher, Inez Januszczak, Natural History Museum Genome Acquisition Lab, Darwin Tree of Life Barcoding collective, Wellcome Sanger Institute Tree of Life programme, Wellcome Sanger Institute Scientific Operations: DNA Pipelines collective, Tree of Life Core Informatics collective, Darwin Tree of Life Consortium

**Affiliations:** 1 Natural History Museum, London, England, UK

**Keywords:** Gastracanthus pulcherrimus, chalcid wasp, genome sequence, chromosomal, Hymenoptera

## Abstract

We present a genome assembly from an individual female *Gastracanthus pulcherrimus* (a chalcid wasp; Arthropoda; Insecta; Hymenoptera; Pteromalidae). The genome sequence is 1,010.0 megabases in span. Most of the assembly is scaffolded into 5 chromosomal pseudomolecules. The mitochondrial genome has also been assembled and is 24.4 kilobases in length.

## Species taxonomy

Eukaryota; Metazoa; Eumetazoa; Bilateria; Protostomia; Ecdysozoa; Panarthropoda; Arthropoda; Mandibulata; Pancrustacea; Hexapoda; Insecta; Dicondylia; Pterygota; Neoptera; Endopterygota; Hymenoptera; Apocrita; Proctotrupomorpha; Chalcidoidea; Pteromalidae; Pteromalinae; *Gastracanthus*; *Gastracanthus pulcherrimus* (Westwood, 1833) (NCBI:txid2922068).

## Background

With females measuring around 5 mm in length, *Gastracanthus pulcherrimus* is a relatively large species of the chalcid wasp family Pteromalidae. It is also a striking species, slender, with a particularly long pronotum, metallic green hues and, in females, two large dark patches on each fore wing. Like most parasitoid wasps, *G. pulcherrimus* is poorly known but it is fairly easily found in deciduous woodland and has a wide range across Europe ( Noyes, 2019), recorded from the Republic of Ireland, Northern Ireland ( Thuróczy & O’Connor, 2009) and from England, but not yet from Wales or Scotland ( Dale-Skey *et al.*, 2016).

Despite its relative conspicuousness, knowledge about the biology of *G. pulcherrimus* is not extensive. It belongs to the subfamily Trigonoderinae, which all seem to be parasitoids of Coleoptera ( Graham, 1969). *Gastracanthus pulcherrimus* is associated with wood and has been reported as a parasitoid of a *Sphenoptera* species (Coloptera: Buprestidae ( Ghahari & Huang, 2012). Graham (1969) expresses doubt about a supposed rearing from an adult byrrhid beetle. By analogy with related Pteromalidae, *G. pulcherrimus* is presumably an idiobiont ectoparasitoid, i.e., hosts will be permanently paralysed, and the larva develops externally on the host.

The family Pteromalidae, for a long time an unwieldy assemblage of disparate chalcid lineages, has recently been split into many different, hopefully monophyletic families ( Burks *et al.*, 2022). *Gastracanthus* is among ten genera comprising the subfamily Trigonoderinae, itself one of eight subfamilies within the diverse and species rich Pteromalidae family. All previously published genomes belong to species in the subfamily Pteromalinae ( Martinson *et al.*, 2017; Werren *et al.*, 2010).

## Genome sequence report

**Figure 1.**
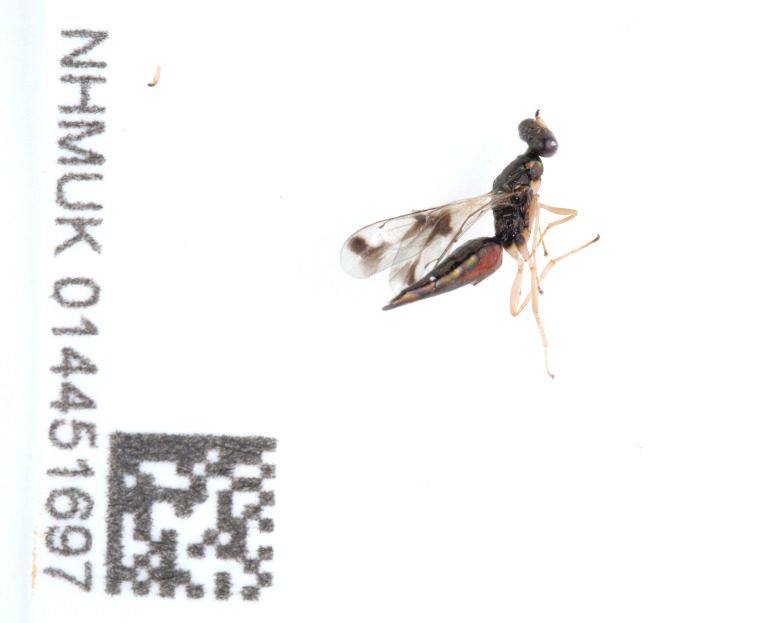
Photograph of the *Gastracanthus pulcherrimus* (iyGasPulc2) specimen used for genome sequencing.

The genome was sequenced from one female *Gastracanthus pulcherrimus* ( Figure 1) collected from Wytham Woods, UK (51.77, –1.31). A total of 27-fold coverage in Pacific Biosciences single-molecule HiFi long reads was generated. Primary assembly contigs were scaffolded with chromosome conformation Hi-C data. Manual assembly curation corrected 48 missing joins or mis-joins and removed one haplotypic duplication, reducing the scaffold number by 6.49%, and increasing the scaffold N50 by 0.45%.

**Figure 2.**
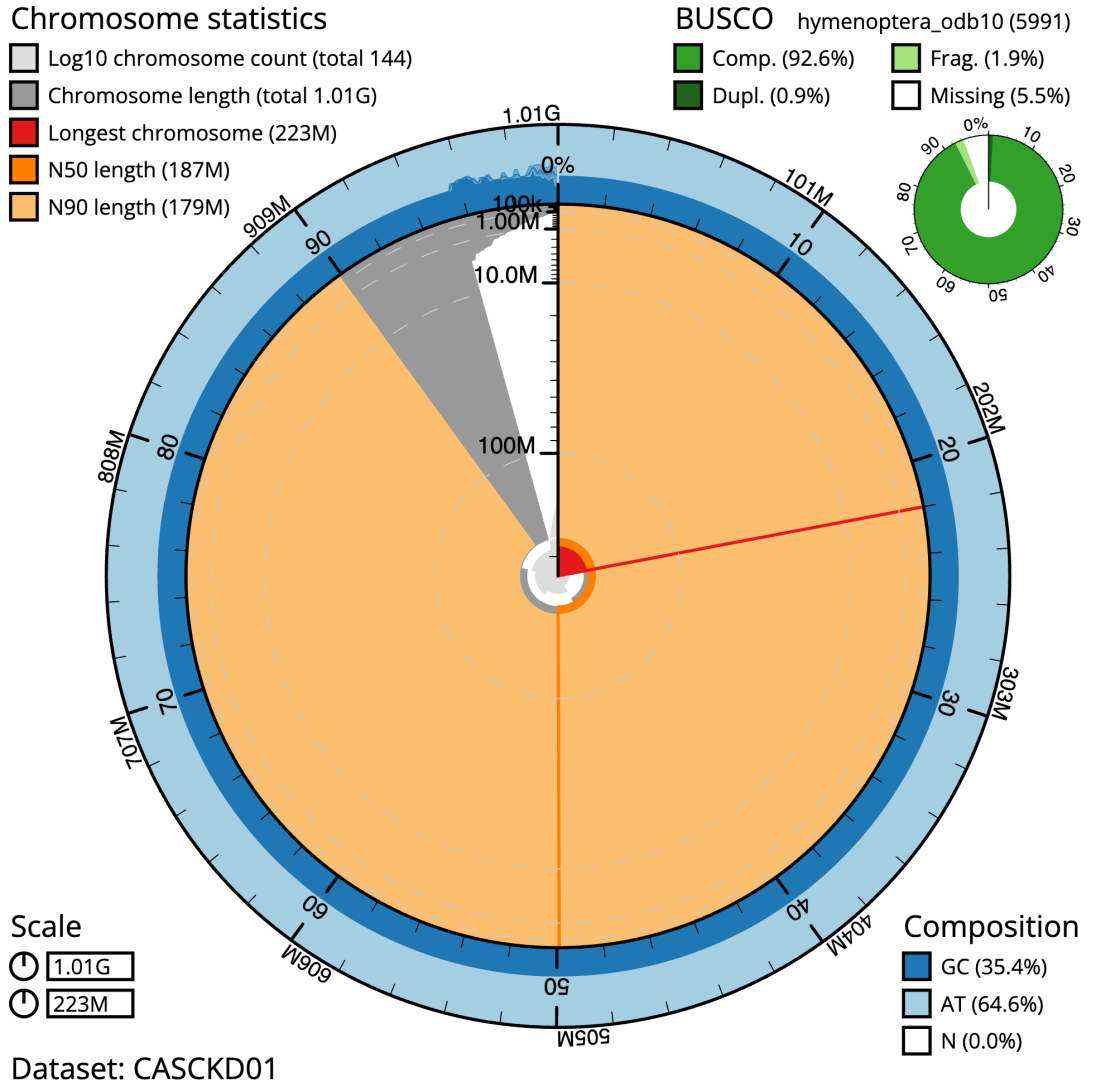
Genome assembly of *Gastracanthus pulcherrimus*, iyGasPulc2.1: metrics. The BlobToolKit Snailplot shows N50 metrics and BUSCO gene completeness. The main plot is divided into 1,000 size-ordered bins around the circumference with each bin representing 0.1% of the 1,010,024,266 bp assembly. The distribution of scaffold lengths is shown in dark grey with the plot radius scaled to the longest scaffold present in the assembly (222,508,353 bp, shown in red). Orange and pale-orange arcs show the N50 and N90 scaffold lengths (186,761,157 and 179,352,141 bp), respectively. The pale grey spiral shows the cumulative scaffold count on a log scale with white scale lines showing successive orders of magnitude. The blue and pale-blue area around the outside of the plot shows the distribution of GC, AT and N percentages in the same bins as the inner plot. A summary of complete, fragmented, duplicated and missing BUSCO genes in the hymenoptera_odb10 set is shown in the top right. An interactive version of this figure is available at https://blobtoolkit.genomehubs.org/view/iyGasPulc2.1/dataset/CASCKD01/snail.

The final assembly has a total length of 1,010.0 Mb in 143 sequence scaffolds with a scaffold N50 of 186.8 Mb ( Table 1). Most (95,67%) of the assembly sequence was assigned to 5 chromosomal-level scaffolds. Chromosome-scale scaffolds confirmed by the Hi-C data are named in order of size ( Figure 2– Figure 5; Table 2). While not fully phased, the assembly deposited is of one haplotype. Contigs corresponding to the second haplotype have also been deposited. The mitochondrial genome was also assembled and can be found as a contig within the multifasta file of the genome submission.

**Table 1.** Genome data for *Gastracanthus pulcherrimus*, iyGasPulc2.1.

Project accession data		
Assembly identifier	iyGasPulc2.1	
Species	*Gastracanthus pulcherrimus*	
Specimen	iyGasPulc2	
NCBI taxonomy ID	2922068	
BioProject	PRJEB59075	
BioSample ID	SAMEA110043171	
Isolate information	iyGasPulc2, female: whole organism (DNA sequencing) iyGasPulc1: whole organisms (Hi-C scaffolding)	
Assembly metrics *		*Benchmark*
Consensus quality (QV)	54.3	*≥ 50*
*k*-mer completeness	99.98%	*≥ 95%*
BUSCO **	C:92.6%[S:91.7%,D:0.9%], F:1.9%,M:5.5%,n:5,991	*C ≥ 95%*
Percentage of assembly mapped to chromosomes	95.67%	*≥ 95%*
Sex chromosomes	-	*localised homologous pairs*
Organelles	Mitochondrial genome assembled	*complete single alleles*
Raw data accessions		
PacificBiosciences SEQUEL II	ERR10798431	
Hi-C Illumina	ERR10802452	
Genome assembly		
Assembly accession	GCA_949152435.1	
*Accession of alternate * *haplotype*	GCA_949152415.1	
Span (Mb)	1,010.0	
Number of contigs	954	
Contig N50 length (Mb)	1.9	
Number of scaffolds	143	
Scaffold N50 length (Mb)	186.8	
Longest scaffold (Mb)	222.5	

* Assembly metric benchmarks are adapted from column VGP-2020 of “Table 1: Proposed standards and metrics for defining genome assembly quality” from ( Rhie *et al.*, 2021). ** BUSCO scores based on the hymenoptera_odb10 BUSCO set using v5.3.2. C = complete [S = single copy, D = duplicated], F = fragmented, M = missing, n = number of orthologues in comparison. A full set of BUSCO scores is available at https://blobtoolkit.genomehubs.org/view/iyGasPulc2.1/dataset/CASCKD01/busco.

**Figure 3.**
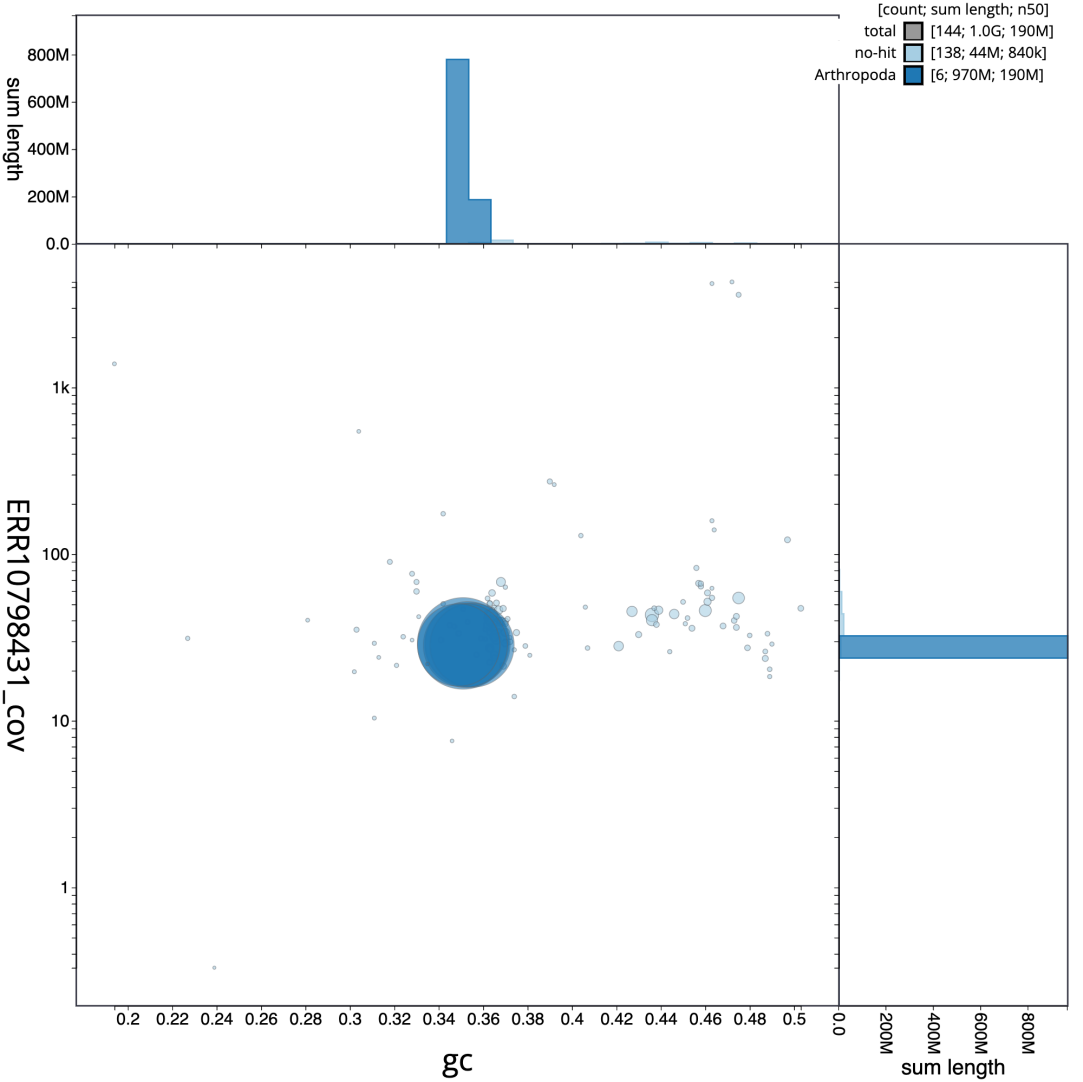
Genome assembly of *Gastracanthus pulcherrimus*, iyGasPulc2.1: BlobToolKit GC-coverage plot. Scaffolds are coloured by phylum. Circles are sized in proportion to scaffold length. Histograms show the distribution of scaffold length sum along each axis. An interactive version of this figure is available at https://blobtoolkit.genomehubs.org/view/iyGasPulc2.1/dataset/CASCKD01/blob.

**Figure 4.**
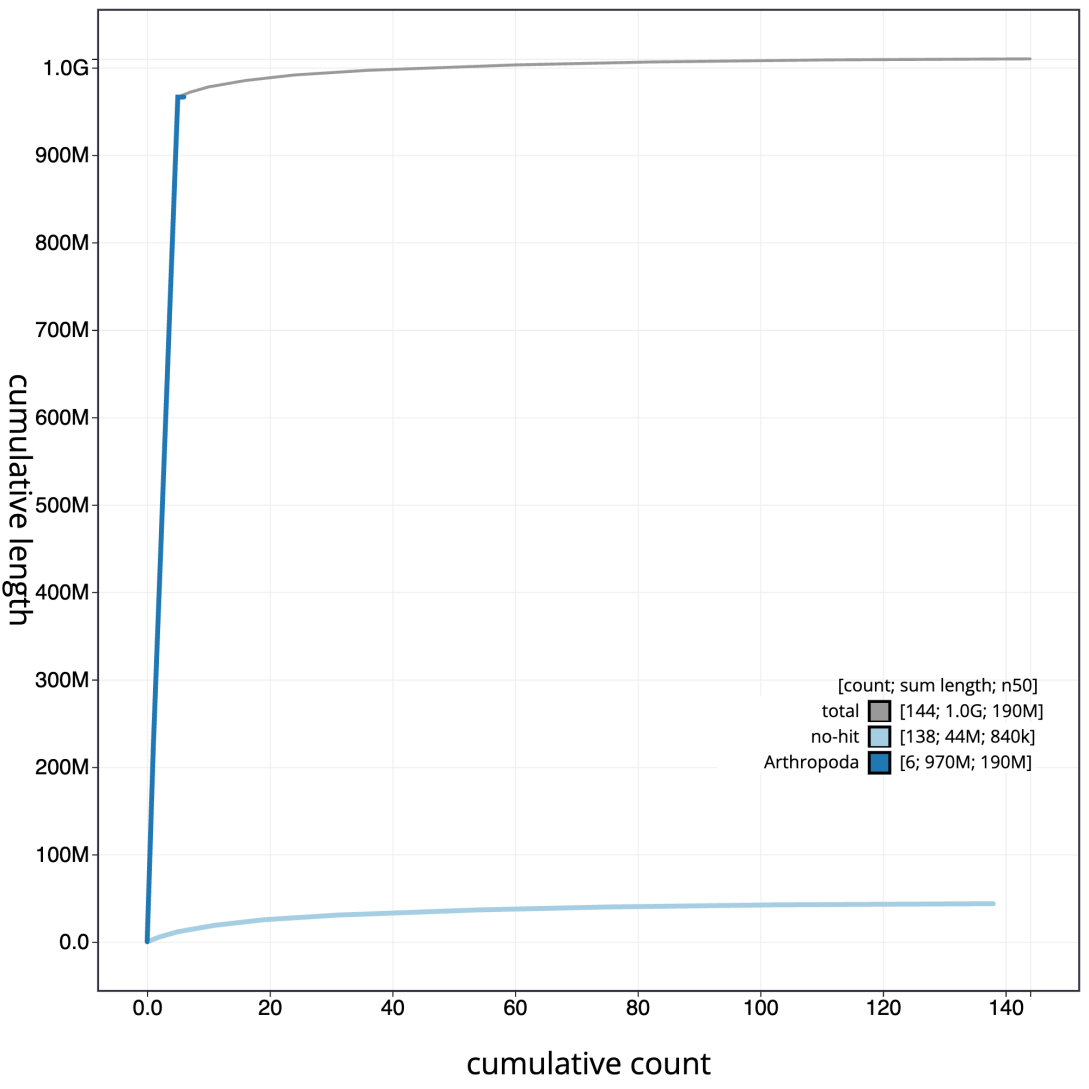
Genome assembly of *Gastracanthus pulcherrimus*, iyGasPulc2.1: BlobToolKit cumulative sequence plot. The grey line shows cumulative length for all scaffolds. Coloured lines show cumulative lengths of scaffolds assigned to each phylum using the buscogenes taxrule. An interactive version of this figure is available at https://blobtoolkit.genomehubs.org/view/iyGasPulc2.1/dataset/CASCKD01/cumulative.

**Figure 5.**
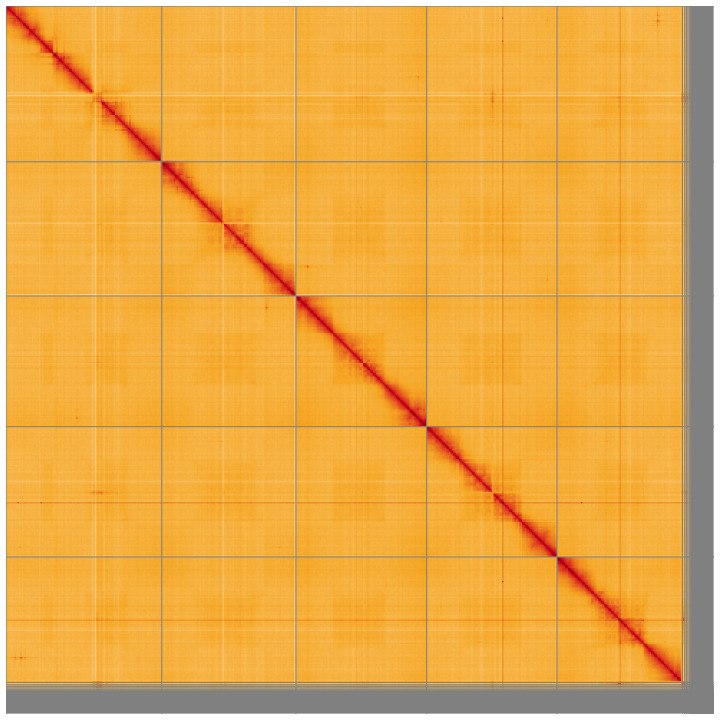
Genome assembly of *Gastracanthus pulcherrimus*, iyGasPulc2.1: Hi-C contact map of the iyGasPulc2.1 assembly, visualised using HiGlass. Chromosomes are shown in order of size from left to right and top to bottom. An interactive version of this figure may be viewed at https://genome-note-higlass.tol.sanger.ac.uk/l/?d=Yrs3ERn6RL-maDBtEFqvcA.

**Table 2.** Chromosomal pseudomolecules in the genome assembly of *Gastracanthus pulcherrimus*, iyGasPulc2.

INSDC accession	Chromosome	Length (Mb)	GC%
OX424572.1	1	222.51	35.0
OX424573.1	2	191.63	35.5
OX424574.1	3	186.76	35.5
OX424575.1	4	186.09	35.0
OX424576.1	5	179.35	35.0
OX424577.1	MT	0.02	19.5

The estimated Quality Value (QV) of the final assembly is 54.3 with *k*-mer completeness of 99.98%, and the assembly has a BUSCO v5.3.2 completeness of 92.6% (single = 91.7%, duplicated = 0.9%), using the hymenoptera_odb10 reference set ( *n* = 5,991).

Metadata for specimens, spectral estimates, sequencing runs, contaminants and pre-curation assembly statistics can be found at https://links.tol.sanger.ac.uk/species/2922068.

## Methods

### Sample acquisition and nucleic acid extraction

Two adult *Gastracanthus pulcherrimus* were collected from Bert’s Pheasant Pen, Wytham Woods, Oxfordshire (biological vice-county Berkshire), UK (latitude 51.77, longitude –1.31) on 2021-09-02, using a light trap. The collectors were Gavin Broad, Chris Fletcher and Inez Januszczak (Natural History Museum). The specimens were identified by Gavin Broad and then dry-frozen at –80°C. The specimen used for DNA sequencing was specimen ID NHMUK014451697, ToLID iyGasPulc2, while specimen ID NHMUK014451655, ToLID iyGasPulc1 was used for Hi-C scaffolding.

DNA was extracted at the Tree of Life laboratory, Wellcome Sanger Institute (WSI). The iyGasPulc2 sample was weighed and dissected on dry ice with tissue set aside for Hi-C sequencing. Tissue from the whole organism was disrupted using a Nippi Powermasher fitted with a BioMasher pestle. High molecular weight (HMW) DNA was extracted using the Qiagen MagAttract HMW DNA extraction kit. HMW DNA was sheared into an average fragment size of 12–20 kb in a Megaruptor 3 system with speed setting 30. Sheared DNA was purified by solid-phase reversible immobilisation using AMPure PB beads with a 1.8X ratio of beads to sample to remove the shorter fragments and concentrate the DNA sample. The concentration of the sheared and purified DNA was assessed using a Nanodrop spectrophotometer and Qubit Fluorometer and Qubit dsDNA High Sensitivity Assay kit. Fragment size distribution was evaluated by running the sample on the FemtoPulse system.

### Sequencing

Pacific Biosciences HiFi circular consensus DNA sequencing libraries were constructed according to the manufacturers’ instructions. DNA sequencing was performed by the Scientific Operations core at the WSI on a Pacific Biosciences SEQUEL IIe (HiFi) instrument. Hi-C data were also generated from whole organism tissue of iyGasPulc1 using the Arima2 kit and sequenced on the Illumina NovaSeq 6000 instrument.

### Genome assembly, curation and evaluation

Assembly was carried out with Hifiasm ( Cheng *et al.*, 2021) and haplotypic duplication was identified and removed with purge_dups ( Guan *et al.*, 2020). The assembly was then scaffolded with Hi-C data ( Rao *et al.*, 2014) using YaHS ( Zhou *et al.*, 2023). The assembly was checked for contamination and corrected using the gEVAL system ( Chow *et al.*, 2016) as described previously ( Howe *et al.*, 2021). Manual curation was performed using gEVAL, HiGlass ( Kerpedjiev *et al.*, 2018) and Pretext ( Harry, 2022). The mitochondrial genome was assembled using MitoHiFi ( Uliano-Silva *et al.*, 2022), which runs MitoFinder ( Allio *et al.*, 2020) or MITOS ( Bernt *et al.*, 2013) and uses these annotations to select the final mitochondrial contig and to ensure the general quality of the sequence.

A Hi-C map for the final assembly was produced using bwa-mem2 ( Vasimuddin *et al.*, 2019) in the Cooler file format ( Abdennur & Mirny, 2020). To assess the assembly metrics, the *k*-mer completeness and QV consensus quality values were calculated in Merqury ( Rhie *et al.*, 2020). This work was done using Nextflow ( Di Tommaso *et al.*, 2017) DSL2 pipelines “sanger-tol/readmapping” ( Surana *et al.*, 2023a) and “sanger-tol/genomenote” ( Surana *et al.*, 2023b). The genome was analysed within the BlobToolKit environment ( Challis *et al.*, 2020) and BUSCO scores ( Manni *et al.*, 2021; Simão *et al.*, 2015) were calculated.

Table 3 contains a list of relevant software tool versions and sources.

**Table 3.** Software tools: versions and sources.

Software tool	Version	Source
BlobToolKit	4.1.5	https://github.com/blobtoolkit/blobtoolkit
BUSCO	5.3.2	https://gitlab.com/ezlab/busco
gEVAL	N/A	https://geval.org.uk/
Hifiasm	0.16.1-r375	https://github.com/chhylp123/hifiasm
HiGlass	1.11.6	https://github.com/higlass/higlass
Merqury	MerquryFK	https://github.com/thegenemyers/MERQURY.FK
MitoHiFi	2	https://github.com/marcelauliano/MitoHiFi
PretextView	0.2	https://github.com/wtsi-hpag/PretextView
purge_dups	1.2.3	https://github.com/dfguan/purge_dups
sanger-tol/genomenote	v1.0	https://github.com/sanger-tol/genomenote
sanger-tol/readmapping	1.1.0	https://github.com/sanger-tol/readmapping/tree/1.1.0
YaHS	1.2a	https://github.com/c-zhou/yahs

### Wellcome Sanger Institute – Legal and Governance

The materials that have contributed to this genome note have been supplied by a Darwin Tree of Life Partner. The submission of materials by a Darwin Tree of Life Partner is subject to the **‘Darwin Tree of Life Project Sampling Code of Practice’**, which can be found in full on the Darwin Tree of Life website here. By agreeing with and signing up to the Sampling Code of Practice, the Darwin Tree of Life Partner agrees they will meet the legal and ethical requirements and standards set out within this document in respect of all samples acquired for, and supplied to, the Darwin Tree of Life Project. 

Further, the Wellcome Sanger Institute employs a process whereby due diligence is carried out proportionate to the nature of the materials themselves, and the circumstances under which they have been/are to be collected and provided for use. The purpose of this is to address and mitigate any potential legal and/or ethical implications of receipt and use of the materials as part of the research project, and to ensure that in doing so we align with best practice wherever possible. The overarching areas of consideration are:

•   Ethical review of provenance and sourcing of the material

•   Legality of collection, transfer and use (national and international) 

Each transfer of samples is further undertaken according to a Research Collaboration Agreement or Material Transfer Agreement entered into by the Darwin Tree of Life Partner, Genome Research Limited (operating as the Wellcome Sanger Institute), and in some circumstances other Darwin Tree of Life collaborators.

## Data Availability

European Nucleotide Archive: *Gastracanthus pulcherrimus*. Accession number PRJEB59075; https://identifiers.org/ena.embl/PRJEB59075. ( Wellcome Sanger Institute, 2023) The genome sequence is released openly for reuse. The *Gastracanthus pulcherrimus* genome sequencing initiative is part of the Darwin Tree of Life (DToL) project. All raw sequence data and the assembly have been deposited in INSDC databases. The genome will be annotated using available RNA-Seq data and presented through the Ensembl pipeline at the European Bioinformatics Institute. Raw data and assembly accession identifiers are reported in Table 1.

## Author information

Members of the Natural History Museum Genome Acquisition Lab are listed here: https://doi.org/10.5281/zenodo.4790042.

Members of the Darwin Tree of Life Barcoding collective are listed here: https://doi.org/10.5281/zenodo.4893703.

Members of the Wellcome Sanger Institute Tree of Life programme are listed here: https://doi.org/10.5281/zenodo.4783585.

Members of Wellcome Sanger Institute Scientific Operations: DNA Pipelines collective are listed here: https://doi.org/10.5281/zenodo.4790455.

Members of the Tree of Life Core Informatics collective are listed here: https://doi.org/10.5281/zenodo.5013541.

Members of the Darwin Tree of Life Consortium are listed here: https://doi.org/10.5281/zenodo.4783558.
