# Supplementary material for: The genome sequence of a chalcid wasp, Gastracanthus pulcherrimus (Westwood, 1833)
Source: Wellcome Open Res. 2023 Oct 12;8:440. [Version 1] doi: 10.12688/wellcomeopenres.19764.1 (PMC11406124; doi:10.12688/wellcomeopenres.19764.1)
Supplement: Peer review history

# Reviewer report: Reviewer response for version 1

**DOI:** 10.21956/wellcomeopenres.21890.r95768

**Published:** 2024-09-16

**Authors:** Daniel Garcia-Souto

**Affiliations:** 1 Bioquímica, xenética e inmunoloxía, Universidade de Vigo (Ringgold ID: 16784), Vigo, Galicia, Spain

This manuscript presents a chromosome-level genome assembly of *Gastracanthus pulcherrimus*, a species of chalcid wasp in the family Pteromalidae. The study provides significant insights into the genomic structure of this relatively understudied species, with a genome span of 1,010.0 megabases. The assembly was primarily scaffolded into five chromosomal pseudomolecules, covering 95.67% of the genome, which highlights the high degree of completeness in the assembly. The authors also sequenced and assembled the mitochondrial genome, which is 24.4 kilobases in length, potentially indicating expansions or repeats in the control region that would, in my honest opinion, require some validation.

 The manuscript makes a notable contribution to the genomic data of the genus, as this is one of the few genomes from the Pteromalidae family outside the Pteromalinae subfamily. Although the final assembly's BUSCO score is slightly lower than the desired threshold (92.6% completeness), this is still a strong result for a genome of such complexity.

Are sufficient details of methods and materials provided to allow replication by others?

Yes

Is the rationale for creating the dataset(s) clearly described?

Yes

Are the datasets clearly presented in a useable and accessible format?

Yes

Are the protocols appropriate and is the work technically sound?

Yes

Reviewer Expertise:

Genomics, Molecular Cytogenetics, Epigenetics, Karyotyping.

I confirm that I have read this submission and believe that I have an appropriate level of expertise to confirm that it is of an acceptable scientific standard.

# Reviewer report: Reviewer response for version 1

**DOI:** 10.21956/wellcomeopenres.21890.r95766

**Published:** 2024-09-04

**Authors:** Jonathan Berenguer Uhuad Koch

**Affiliations:** 1 USDA-ARS Pollinating Insect Research Unit, Maryland, USA

The article describes a genome assembly of *Gastracanthus pulcherrimus *from a female specimen. The authors clearly articulate the generation of the genome assembly using PacBio HiFi and HiC. The genome assembly still needs to be annotated, and in currently in the queue. 

**One minor point **

- Rephrase "The family Pteromalidae, for a long time an unwieldy assemblage of disparate chalcid lineages, has recently been split into many different, hopefully monophyletic families" to "The family Pteromalidae, for a long time an unwieldy assemblage of disparate chalcid lineages, has recently been split into many different, likely monophyletic families"

Are sufficient details of methods and materials provided to allow replication by others?

Yes

Is the rationale for creating the dataset(s) clearly described?

Yes

Are the datasets clearly presented in a useable and accessible format?

Yes

Are the protocols appropriate and is the work technically sound?

Yes

Reviewer Expertise:

population genetics, genomics

I confirm that I have read this submission and believe that I have an appropriate level of expertise to confirm that it is of an acceptable scientific standard.
